# *In Vitro* Chronic Administration of ERbeta Selective Ligands and Prostate Cancer Cell Growth: Hypotheses on the Selective Role of 3beta-Adiol in AR-Positive RV1 Cells

**DOI:** 10.1155/2014/801473

**Published:** 2014-04-29

**Authors:** Alessandra Colciago, Massimiliano Ruscica, Ornella Mornati, Margherita Piccolella, Marina Montagnani-Marelli, Ivano Eberini, Claudio Festuccia, Paolo Magni, Marcella Motta, Paola Negri-Cesi

**Affiliations:** ^1^Department of Pharmacological and Biomolecular Sciences, University of Milano, Via Balzaretti 9, 20133 Milano, Italy; ^2^Department of Biotechnological and Applied Clinical Sciences, University of L'Aquila, Via Vetoio, Coppito 2, 67100 L'Aquila, Italy

## Abstract

Prostate cancer (PC) progression from androgen-dependent (AD) to castration-resistant (CR) disease is a process caused by modifications of different signal transduction pathways within tumor microenvironment. Reducing cell proliferation, estrogen receptor beta (ERbeta) is emerging as a potential target in PC chemoprevention. Among the known selective ERbeta ligands, 3beta-Adiol, the endogenous ligand in the prostate, has been proved to counteract PC progression. This study compares the effects of chronic exposure (1–12 weeks) to different ERbeta selective ligands (DPN, 8beta-VE2, 3beta-Adiol) on proliferation of human androgen-responsive CWR22Rv1 cells, representing an intermediate phenotype between the AD- and CR-PC. 3beta-Adiol (10 nM) is the sole ligand decreasing cell proliferation and increasing p21 levels. *In vitro* transcriptional activity assays were performed to elucidate different behavior between 3beta-Adiol and the other ligands; in these experiments the endogenous and the main ERbeta subtype activation were considered. It is concluded that ERbeta activation has positive effects also in androgen-responsive PC. The underlying mechanisms are still to be clarified and may include the interplay among different ERbeta subtypes and the specific PC microenvironment. ERbeta agonists might be useful in counteracting PC progression, although the final outcome may depend upon the molecular pattern specific to each PC lesion.

## 1. Introduction

Prostate cancer (PC) represents one of the main leading causes of death in men worldwide [1]. This is mainly due to a high rate recurrence and progression of the disease to a castration-resistant and disseminated stage (CR-PC), in which therapeutic options are few and often only palliative [1]. Thus, the discovery of drugs able to positively manage CR-PC and/or to delay its appearance still represents an important clinical challenge.

Estrogens, alone or along with androgens, are important players of prostate carcinogenesis and progression. Indeed, chronically high estrogenic levels are associated with increased risk to develop PC. However, anticancer activity has been observed in many instances by using synthetic or herbal-derived estrogens [2–5]. These conflicting observations are possibly due to the presence of two classes of estrogen receptors (ERalpha and ERbeta) [6, 7], which display differences in localization, expression levels, and functional roles in prostate biology and carcinogenesis. ERbeta, which is largely localized in the epithelial compartment, is linked to antiproliferative and differentiating effects [7–12].* In vitro* data have shown how ERbeta-driven inhibitory activity on PC biology might be mediated by induction of apoptosis [12], by enhanced synthesis of cell cycle inhibitor proteins [4, 13], or by a negative regulation of cell adhesion molecules [14]. Furthermore, the loss of ERbeta is associated with the progression from normal prostate epithelium to PC [15]. All these findings point to a major role of ERbeta to protect prostate cells from uncontrolled proliferation and malignant transformation. Thus, ERbeta activation by specific agonists may be a feasible option treatment for PC chemoprevention and CR-PC management. However, the mechanism of action of ERbeta is rather complex and still unclear due to the discovery of at least five ERbeta different isoforms resulting from alternative splicing of the same gene. Among them, ERbeta1, 2, and 5 are the most studied isoforms in human PC. ERbeta1, which is the one primarily lost during PC progression, is defined as the wild-type isoform and it is related to the antiproliferative and the proapoptotic activity [12, 16]. On the contrary, ERbeta2 and ERbeta5 bind estrogens with different affinity (none and low affinity, resp., [17]) and are associated to increased cell proliferation and enhanced cell migration, as well as to a PC poor prognosis [18, 19]. It is suggested that these isoforms, which are often coexpressed with ERbeta1 in many tissues, including the prostate [16, 17, 20, 21], bind as homo- or heterodimers to canonical ERE sequences and act as variable parameters with enhancer or dominant negative functions [17, 18, 20, 22]. Moreover, coexpression of ERbeta1 with ERbeta2 or ERbeta5 in HEK293 cells significantly enhances ERE-mediated transactivation when activated by estradiol or phytoestrogens [17]. To our knowledge, the ability of these complexes to activate transcription upon binding with ERbeta selective agonists has never been evaluated.

Thanks to the significant differences in the ligand binding domain between ERalpha and ERbeta, a series of ERbeta selective agonists have been developed in these last years [5, 23, 24], and most of them have been also tested for their biological activity in different experimental models [25–28]. The various ERbeta selective agonists have the same transcriptional activity on a battery of genes; however, it is demonstrated that they may also display gene-specific activation/repression resulting in distinct biological outcomes and possibly clinical effects [29]. The fact that different ERbeta-selective agonists might elicit different biological results points to the need of a careful evaluation of diverse structural classes of compounds in various disease models to identify the optimal ERbeta agonist in each condition.

Owing to the largely planar configuration of the phytoestrogens (the first ERbeta selective ligands discovered), nonsteroidal compounds retaining a similar topology have been synthesized; among these, diarylpropionitrile (DPN) is considered the prototype molecule of the group [5, 23]. However, compounds with nonplanar rigid configuration have shown a more robust and greater selectivity for ERbeta than the planar ones [5]. The prototype of this latter group is 8vinylestrane-1,3,5(10)-triene-3,17beta-diol (8beta-VE2) [5]. High affinity for ERbeta is also displayed by 5alpha-androstane-3beta,17beta-diol (3beta-Adiol), an endogenous metabolite of DHT which classically does not bind to the wild-type AR [30]. As the intraprostatic levels of 3beta-Adiol* in vivo* are about 100-folds higher than those of estradiol, this steroid is considered the natural ligand of ERbeta in the gland [11].

Among the different available* in vitro* PC models, the CWR22RV1 (Rv1) cells, which are derived from a primary androgen-dependent human PC tumor (CWR22) orthotopically transplanted in castrated nude mice, represent an intermediate phenotype between the AR-dependent and castration-resistant tumor. Rv1 cells are androgen ablation-resistant, but still androgen-responsive [31, 32]. They express ARs (both wild-type and mutated forms [33]), but conflicting results are reported on the expression levels of ERalpha and ERbeta [12, 34]. Due to the 35/40-h doubling time, they represent a suitable cell-based model, resembling the “*in vivo*” condition, to study the effect of a long-lasting treatment on cell proliferation [31].

The aim of the study was to evaluate in Rv1 cell line; (a) the expression of the different isoforms of androgen and estrogen receptors; (b) the effects driven by a chronic exposure to DPN, 8beta-VE2, and 3beta-Adiol on cell proliferation rate, on the expression of AR and ER gene levels and on the expression of some proteins involved in cell cycle arrest (PTEN, p21 and cyclin E). A series of* in vitro* transcriptional activity assessments were then performed to elucidate the different behavior between 3beta-Adiol and the other ERbeta selective ligands in Rv1 cell.

## 2. Material and Methods

### 2.1. Chemicals and Plasmids

2,3-bis(4-hydroxyphenyl)-propionitrile (DPN, Tocris Cookson, Ellisville, MO, USA), 8vinylestrane-1,3,5(10)-triene-3,17beta-diol (8beta-VE2, kindly provided by Dr. K. Prelle, Bayer Schering Pharma AG), and 5alpha-androstane-3beta,17beta-diol (3beta-Adiol, Sigma-Aldrich, Milano, Italy) were used as ERbeta selective agonists. ICI 182,780 (Tocris Cookson, Ellisville, MO, USA) was used as estrogen receptor antagonist. All compounds were dissolved in ethanol.

pCMV5-ERbeta1, pCMV5-ERbeta2, pCMV5-ERbeta5, and pCMV5-EMPTY were kindly provided by Dr. P. G. V. Martini, Shire HGT, Boston, MA; pGL3-2ERE-pS2-luc was kindly provided by Dr. M. Marino, Rome, and pgL 4.0 hRLuc was from Promega (Milano, Italy).

### 2.2. Cell Cultures and Treatments

CWR22Rv1 (Rv1) cells were originally obtained from DSMZ (Frankfurt, Germany); HEK293 cell line was originally obtained by American Type Culture Collection (Rockville, MD) and currently used in our laboratory.

All cell culture reagents were purchased from Biochrom (Biochrom KG, Berlin, Germany). Rv1 cells were routinely grown at 37°C in a humidified atmosphere (5% CO_2_—95% air) in 100 mm Petri dishes in phenol red free RPMI 1640 supplemented with 5% of heat inactivated fetal calf serum (FCS, GIBCO), glutamine (2 mM), penicillin (100 IU/mL), and streptomycin (100 microg/mL). Medium was changed biweekly. HEK293 were routinely maintained in the same culture conditions in 10% FCS phenol red free RPMI 1640.

### 2.3. Experimental Schedule in Long-Term Experiments

**Figure 1:**
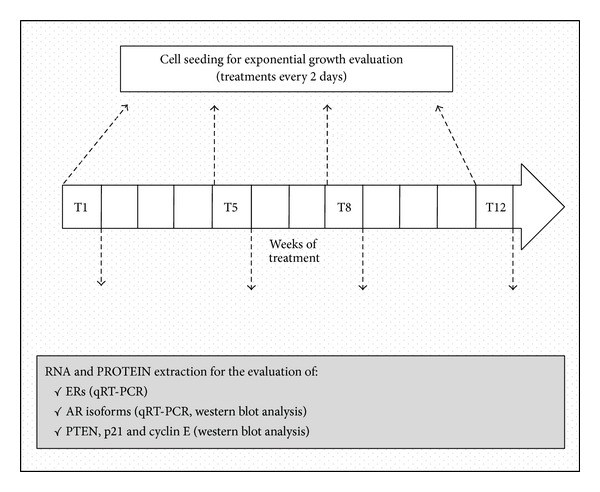
Experimental schedule of chronic treatment.

Rv1 cells were seeded in 100 mm Petri dishes in phenol-red free RPMI 1640 supplemented with 5% charcoal stripped-FCS (FCS-CH) (2 independent samples/treatments/times) and chronically treated every 2 days with ethanol (control cells), DPN, 8beta-VE2, or 3beta-Adiol (all 10 nM) up to 12 weeks, on a weekly propagation schedule. At the beginning of the long-term exposure (T1) and after 5, 8, and 12 weeks (T5–T12) of chronic treatment, part of the cells from each group was harvested and utilized for RNA/protein extraction and for the growth rate evaluation. The schedule of chronic treatments is outlined in Figure 1.

### 2.4. Growth Rate Evaluation

Cells from the chronic exposure were seeded in 100 mm Petri dishes in 5% FCS-CH phenol-red free RPMI 1640 (3 independent samples/treatments/times) and the corresponding treatment went on every 2 days for 10 days. After 3, 5, 7, and 10 days, cells from some of the Petri dishes were harvested and counted by Tripan blue exclusion in a Burker chamber (see Figure 1).

### 2.5. Real-Time PCR

Total RNA from control and treated cells was extracted by the phenol-chloroform method according to standard protocols [35] and used for real-time PCR (qPCR). A mean of 2 independent RNA samples was used for each determination. Reverse transcription was performed on 1 *μ*g of total RNA from each sample according to the manufacturer's protocol (iScript cDNA synthesis kit, BioRad, Segrate, Italy) using random primers. qPCR was done in singleplex in CFX96 Touch Real-Time PCR Detection System (BioRad, Segrate, Italy) using two different experimental protocols: ERalpha, ERbeta and AR genes were amplified using the SsoFast Probes supermix (BioRad, Segrate, Italy) and specific assays on demand (AoD, Life Technologies, Monza, Italy); ERbeta isoforms were amplified using the SsoAdvanced SYBR Green SuperMix (BioRad, Segrate, Italy) and the specific sets of primers listed in Table 1, designed using the Primer 3 software and purchased by Sigma Aldrich, Milano, Italy.

Each sample was analysed in triplicate. Relative mRNA levels were calculated using the comparative CT method (2^−ΔΔCt^).

### 2.6. Western Blot Analysis

Constitutive proteins from control and treated cells were prepared by lysing in RIPA buffer with proteases inhibitors. Total proteins extracts (30 microgr/sample), determined with BCA assay (Pierce, Rockford, IL, USA), were resolved on SDS-PAGE followed by electrotransfer onto nitrocellulose membrane. The fluorescent qDot system (Life Technologies Italia, Monza, Italy) was used for ERbeta detection; the enhanced chemiluminescence (ECL) detection kit (GE Healthcare, Milano, Italy) was used for AR, PTEN, and p21 detection. Specific primary and secondary antibodies are listed in Table 2.

### 2.7. Transient Transfections and Transcriptional Activity Assay

Rv1 or HEK-293 cells, plated in 96-wells plate and maintained in RPMI 1640 without phenol-red and FCS, have been transfected by using Lipofectamine 2000 (Life Technologies Italia, Monza, Italy). Transfection was performed using a total of 0.2 microgr of plasmid DNAs/well, according to manufacturer's protocol. After 6 hours, the transfection medium was replaced with RPMI 1640 without phenol-red with 5% (Rv1) or 10% (HEK 293) FCS-CH containing the appropriate treatment. Transcriptional activity was evaluated 22 hours later by the luciferase assay (DUAL-GLO Luciferase Assay System kit, Promega, Milano, Italy), according to the manufacturer's protocol. The inducible firefly luciferase activity has been normalized by renilla luciferase. Each sample was assessed in duplicate.

### 2.8. Statistical Analysis

The statistical analysis of the row data was performed by one-way parametric ANOVA and expressed as mean ± SD; post hoc analyses were performed by Tukey's Multiple Comparison Test, using the Graph-Pad software for Macintosh (Evanston, IL). Only* P* values < 0.05 were considered statistically significant.

Cell growth rate was analyzed by an exponential curve fitting computer program (ESPSS) followed by the statistical analysis of the fitted curve parameters through parametric ANOVA and by the Student-Newman-Keuls post hoc Test for multiple comparisons. Only* P* values < 0.05 were considered statistically significant.

## 3. Results

### 3.1. Steroid Hormone Receptor Pattern in Rv1 Cells

**Figure 2:**
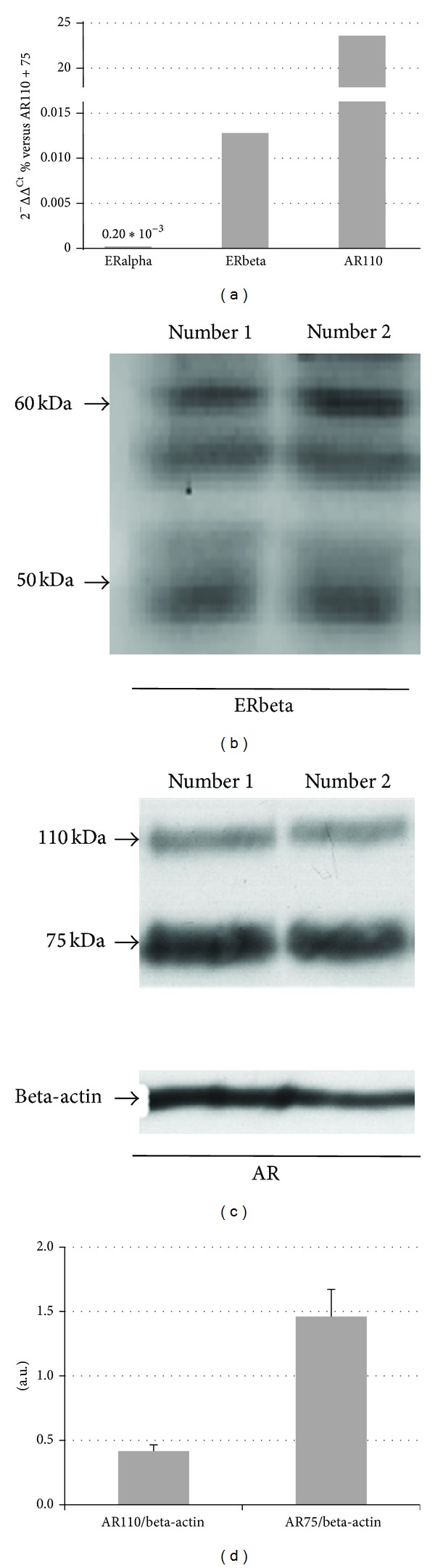
Relative expression of ERs and ARs by qPCR (a); Western blot analysis of ERbeta (b) and ARs (c); densitometric analysis of ARs (d).

Due to conflicting data on ERbeta expression in Rv1 cells, first of all we have assessed the presence of the endogenous ERs, along with that of ARs in our experimental model (Figure 2). Panel (a) shows the expression pattern of ERalpha, ERbeta and of the 110 kDa form of AR, evaluated by qPCR and expressed as % versus the 110 + 75 AR transcripts (AR total) after normalization for the housekeeping gene HPRT. It is apparent from the panel that this cell line expresses both ERalpha and ERbeta. Even though the levels of ERbeta are higher than those of ERalpha, taken as a whole, the expression of the two ER is very low in comparison to that of ARs; the 110 kDa AR represents roughly 20% of the totality of AR transcripts. In this qPCR experiment AR75 was not evaluated separately due to the impossibility to design a set of specific primers.

To confirm the presence of ERbeta also at protein level, Western blot analysis was performed on two independent RV1 samples using an antibody that maps to the N-terminus (common to all the ERbeta subtypes, see below).  Figure 2(b) shows the presence of at least three immunoreactive bands with a MW within the 50–60 kDa range, which might correspond to the three main ERbeta subtypes present in CP cells [17]. AR75 and AR110 expression levels were evaluated separately by Western blot analysis. A representative Western blot carried out using a polyclonal antibody directed against the amino-terminus of the protein, which recognizes all the different AR forms, is shown in panel (c). It is evident that Rv1 cells contain both the AR form of 110 kDa (considered the wild type) and that of 75–80 kDa, corresponding to some forms truncated at the carbossi-terminal; panel (d) reports the mean ± SD of the densitometric analysis of a series of samples after normalization with actin. As it appears from the figure, and in line with the qPCR results, the total amount of the truncated forms is about threefold higher than that of AR110, which represent the 20% of the total AR levels.

### 3.2. Determination of the Optimal Dose for the Chronic Studies

**Figure 3:**
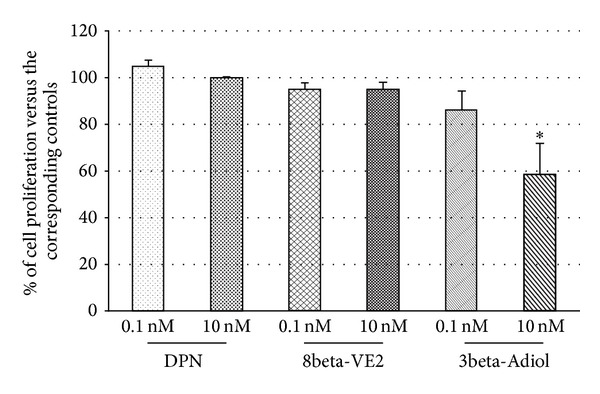
Dose-response effect of ERbeta selective agonists on short-term (9 days) proliferation of Rv1 cells. Data are mean ± SD and are expressed as percent of the corresponding controls. *P < 0.05 versus 3beta-Adiol 0.1 nM.

Previous preliminary experiments performed in our laboratory using other AR-PC cell lines showed that both DPN and 8beta-VE2 at the concentration of 10 nM were able to significantly reduce DU145 cell proliferation after 9 days of exposure (data not shown). Thus, to test the effect of DPN, 8beta-VE2, and 3beta-Adiol specifically on Rv1 cell proliferation, the same (10 nM) or a hundred times lower (0.1 nM) dose of each drug has been administered in a 9-day treatment schedule (Figure 3).

To compare experiments carried out in different times, the data in the figure are expressed as percent versus their own control. As shown, none of the three ligands is effective at the lower dose. Only 3beta-Adiol is able to significantly decrease cell proliferation at the dose of 10 nM, being the same dose of DPN and 8beta-VE2 ineffective in this cell line. Even though the two latter compounds are ineffective after a 9-day exposure, no higher doses have been tested because of the possibility of cross-activation of ERalpha [23, 36], or the achievement of the 100% of the transcriptional activity, as in the case of 8beta-VE2 [37].

### 3.3. Rv1 Proliferation Rate during Chronic Exposure

**Figure 4:**
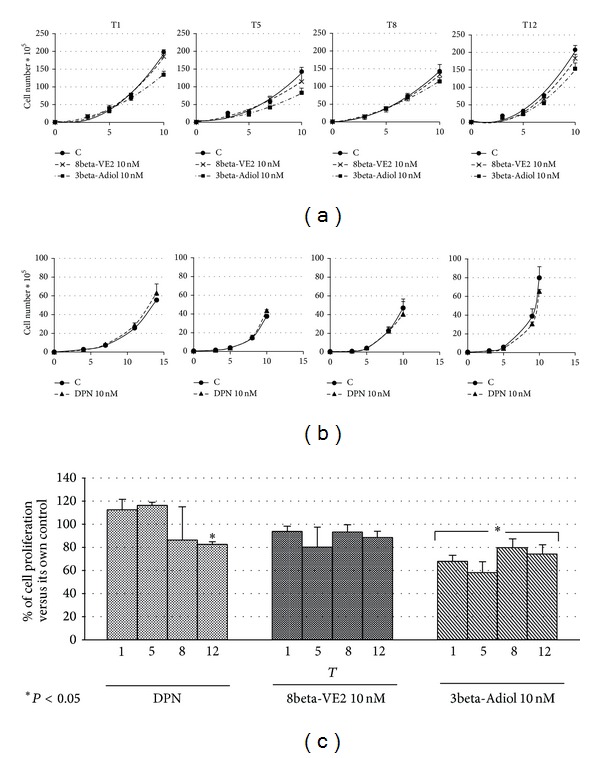
Effect of chronic exposure to ERbeta selective ligands on Rv1 cell proliferation: proliferation curves evaluated at T1-5-8-12 with vehicle (C), 8betaVE2, or 3beta-Adiol (a); with vehicle (C) and DPN (b). Cumulative data of the relative proliferation rate during the chronic treatments (c): data are mean ± SD and are expressed as percent of the corresponding controls.  *P < 0.05 versus the corresponding controls.

The proliferation rate of Rv1 chronically exposed to 10 nM DPN, 8beta-VE2, or 3beta-Adiol has been assessed in two separate sets of experiments. The corresponding exponential curves calculated at different weeks during the treatment are reported in Figures 4(a) and 4(b).

When tests for multiple comparisons have been applied to the fitted curve parameters, no statistically significant differences have been detected among the curves for all compounds at all the time points examined. However, in the case of cells exposed to 3beta-Adiol a constant decrement is apparent at all the time frames (Figure 4(a)). The statistical comparison of the last point of each curve only (10 days of exposure) for each treatment by a restricted ANOVA analysis shows statistically significant differences in comparison to control cells at all the time frames (from T1 to T12) for 3beta-Adiol, while the chronic treatment with 8beta-VE2 results in a slight but not significant decrease of cell proliferation (Figure 4(c)). The same figure shows that the 10 nM DPN is completely ineffective, but after 12 weeks of chronic exposure, when a significant antiproliferative effect is apparent (Figure 4(c)). In our experimental conditions, the efficacy of 3beta-Adiol to slow cell proliferation is also supported by the increase of the doubling time calculated for the proliferation curves at each time frame in comparison to the corresponding control cells (from 48–60 h of controls to 65–74 h of 3beta-Adiol treated cells).

### 3.4. Influence of the Chronic Exposure on Cell Cycle Regulators and on AR and ERbeta Levels

**Figure 5:**
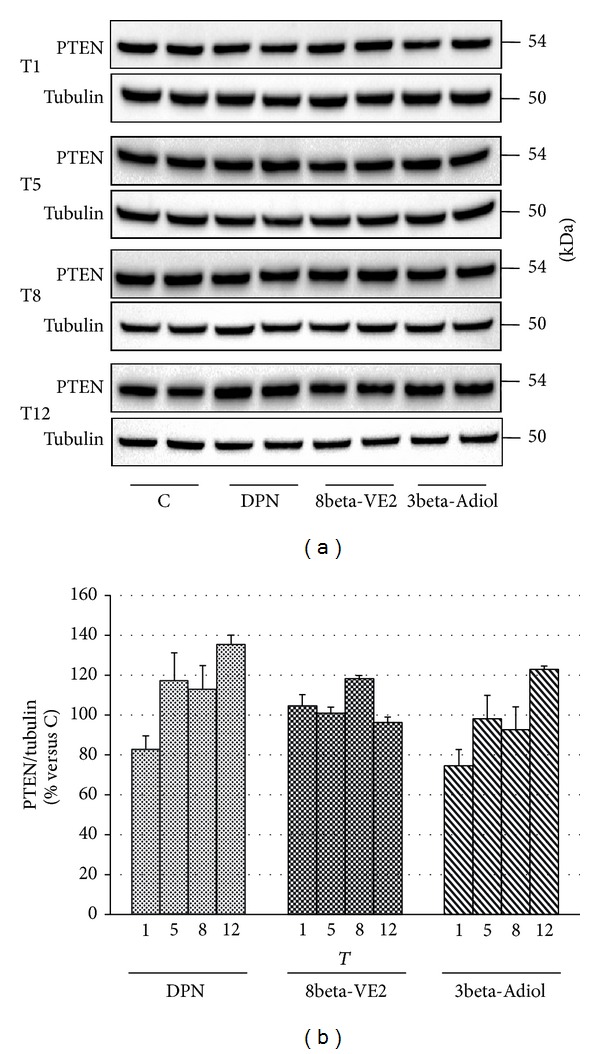
Effects of different chronic treatments with DPN, 8beta-VE2, and 3beta-Adiol on PTEN protein expression in Rv1 cells: immunoreactive bands (representative Western Blot) during chronic treatments (from T1 to T12) (a); histograms representing the time course of PTEN protein expression, normalized to the levels of tubulin, grouped by each treatment; values are expressed as mean ± SD (b).

**Figure 6:**
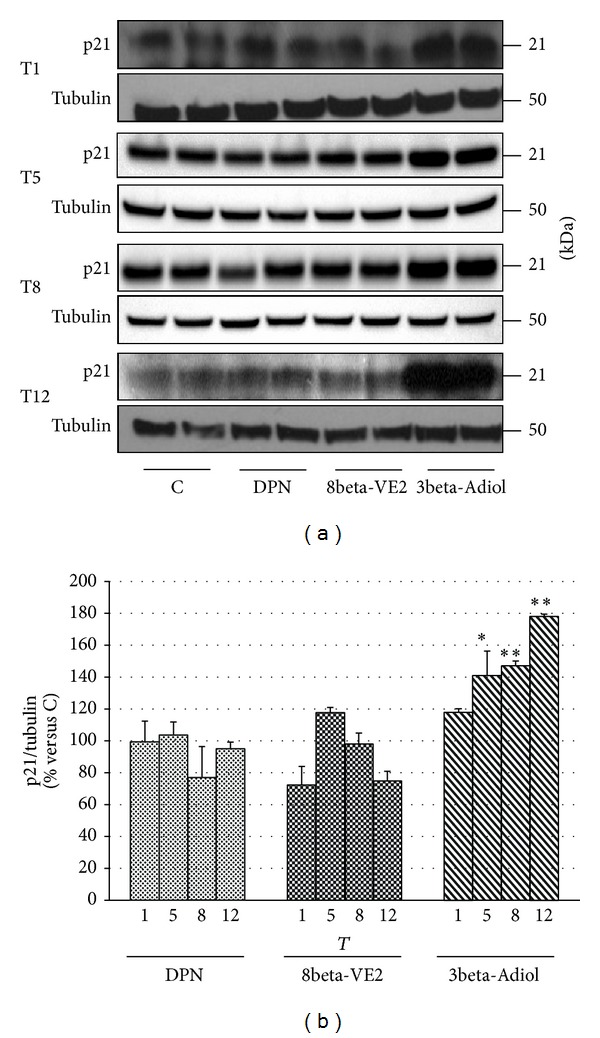
Effects of different chronic treatments with DPN, 8beta-VE2, and 3beta-Adiol on p21 protein expression in Rv1 cells: immunoreactive bands (representative Western Blot) during chronic treatments (from T1 to T12) (a); histograms showing the time course of p21 protein expression, normalized to the levels of tubulin, grouped by each treatment; data are expressed as mean ± SD;  *P < 0.05 versus corresponding controls;  **P < 0.01 versus corresponding controls (b).

To clarify some of the molecular mechanisms at the basis of the antiproliferative action of the ERbeta selective agonists, we evaluated by Western blot analysis the modifications of PTEN (Figure 5) and of p21 (Figure 6) during the chronic treatment with DPN, 8beta-VE2, and 3beta-Adiol. In both figures, panels (a) show representative Western blots of PTEN and p21 levels in cells exposed to the three drugs from T1 to T12, respectively, while in panels (b) the cumulative results of different experiments have been pooled together as a function of the treatment and expressed as fold variation in comparison to the corresponding control samples. As far as PTEN is concerned, the results show a slight and not significant increase of PTEN levels in the DPN-treated cells (35% at T12), while neither 8beta-VE2 nor 3beta-Adiol is able to consistently enhance the expression of this cell cycle regulator (Figure 5(b)). On the contrary, as clearly appears from the Figure 6(b), only 3beta-Adiol leads to a progressive significant increase in p21 protein expression (+41%, *P* < 0.05; +47%, *P* < 0.01, and +78%  *P* < 0.01 versus control, from T5 to T12). Treatments with DPN or 8beta-VE2 are completely ineffective. In parallel, only 3beta-Adiol induces a decrease of cyclin E expression levels (data not shown).

Neither 3beta-Adiol nor the other ERbeta selective agonists are able to influence the expression levels of AR and ERbeta, as revealed by qPCR experiments (data not shown).

### 3.5. Transcriptional Activity of Selective ERbeta Agonists

**Figure 7:**
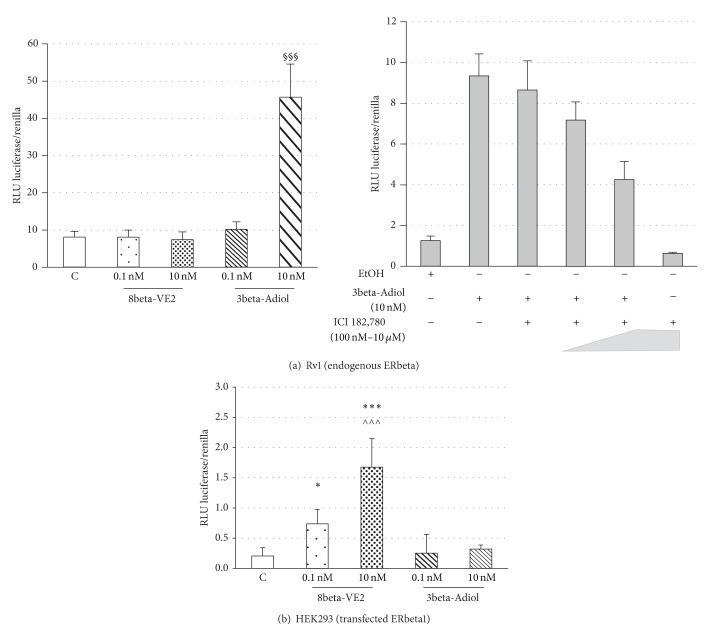
Transcriptional activity of ERbeta agonists in Rv1 (a) and in HEK 293 (b) cells: data are expressed as mean ± SD of the ratio between the luminescence (RLU) of the experimental over the control reporter;  *P < 0.05 versus C and 3beta-Adiol 1 and 10 nM;  ***P < 0.001 versus C and 3beta-Adiol 1 and 10 nM; ^§§§^ *P* < 0.001 versus C and 8beta-VE2 1 and 10 nM; ^∧∧∧^ *P* < 0.001 versus 8beta-VE2 1 nM.

To elucidate the different behavior between 3beta-Adiol and the other ERbeta selective ligands on Rv1 cell growth, first of all we tested the ability of the compounds to activate transcription through the binding of ERbeta to ERE sequences (Figure 7). In this set of experiments, a reporter construct containing 2 estrogen response elements (EREs) coupled to luciferase has been transiently transfected into Rv1 cells. The stimulation of the transfected cells with 10 nM 3beta-Adiol (but not with the 0.1 nM dose) resulted in a huge increase of luciferase activity (about 35-fold, Figure 7(a), left panel). The transcriptional activity of 3beta-Adiol is dose-dependently inhibited by the addition of the pure antiestrogen ICI 182.780 (Figure 7(a), right panel), confirming that Rv1 cells possess an endogenous transcriptionally active ERbeta and that 3beta-Adiol, at the doses used in the proliferation studies, mediates the transcription through EREs. Surprisingly, neither DPN (not shown) nor 8beta-VE2, at the dose of 0.1 and 10 nM, was able to activate the endogenous ERbeta-mediated transcription in Rv1 cells (Figure 7(a), left panel). On the contrary, when the full length ERbeta (ERbeta1) was transiently expressed in HEK293 cells together with the 2ERE-containing gene reporter coupled to luciferase, the exposure to 8beta-VE2 at the doses of 0.1 and 10 nM results in a dose dependent increase of the transcriptional activity, while no response was elicited by the same amounts of 3beta-Adiol (Figure 7(b))

### 3.6. Have the Various ERbeta Isoforms a Role in Determining the Different Activity of 3beta-Adiol and 8beta-VE2?

**Figure 8:**
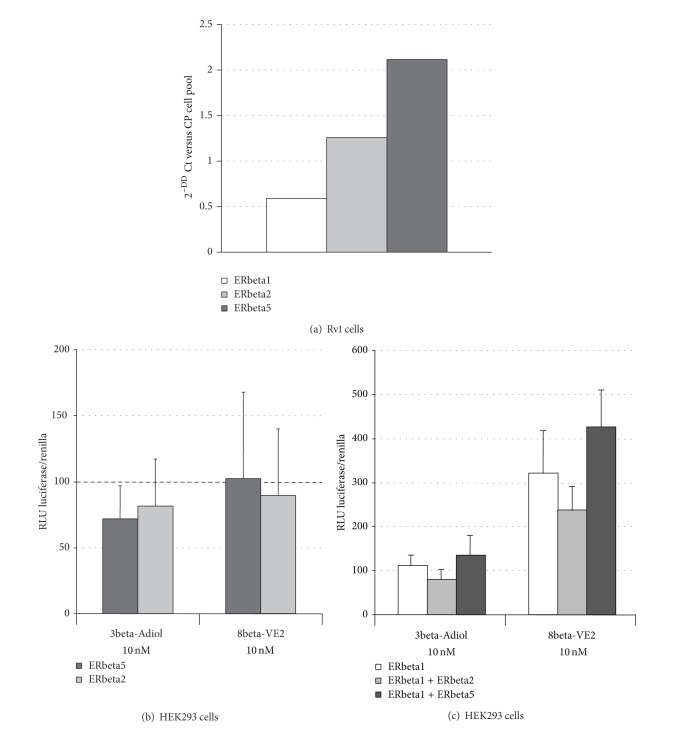
Relative expression of ERbeta isoforms by qPCR in Rv1 cells (a); ERbeta isoform-specific transcriptional activity induced by 3beta-Adiol and 8betaVE2 in HEK293 transfected cells ((b), (c)). ERbeta 2 and ERbeta5 were transfected separately (b) or together with ERbeta1 (c). Data are expressed as mean ± SD of the ratio between the luminescence (RLU) of the experimental over the control reporter.  **P < 0.01 versus beta1 + beta5 (8betaVE2);  ***P < 0.001 versus the corresponding transfected cells in presence of 3beta-Adiol.

To answer this question, we first assess by qPCR the expression pattern of ERbeta subtypes in Rv1 cells in comparison to a mix of RNAs from different AD- and CR-PC cell lines (CWR22, Rv1, PC3, and DU145 cells). The results obtained, shown in Figure 8(a), indicate that Rv1 cells possess low but detectable levels of ERbeta1 and almost three times higher amounts of ERbeta2; ERbeta5 is the most expressed ERbeta subtype (about 5 times more than ERbeta1).

Figure 8(b) shows the results of cotransfection experiments in HEK293 cells, in which the ERE-mediated transcriptional activity of ERbeta1 alone or along with ERbeta2 and ERbeta5 was assessed in presence of 3beta-Adiol or 8beta-VE2. Data are expressed as percent of variation versus control cells transfected with the same plasmids and treated with ethanol. First of all it is possible to note that, in agreement with the previous experiments, 10 nM 8beta-VE2 stimulates transcription both in presence of ERbeta1 alone and in presence of the two hetero-dimers (2- to 4-fold of control cells), while 3beta-Adiol is completely ineffective. Moreover, when activated by 8beta-VE2, the hetero-dimer beta1:beta5 induces a significant increase of the ERE-mediated transcription in comparison to both the homo-dimer beta1:beta1 and the hetero-dimer beta1:beta2. The presence of the ERbeta2 subtype in the hetero-dimer causes a slight but not significant reduction of the ERE-mediated transcriptional activity in comparison to the homo-dimer beta1:beta1.

## 4. Discussion

In the present study we analyzed whether a long-term activation of ERbeta by selective agonists was able to decrease the proliferation of Rv1 PC cells and, if so, which are the underlying molecular mechanisms. In particular, we assessed the effects of DPN [23] and 8beta-VE2 [5] in comparison to the natural ligand 3beta-Adiol [11]; DPN and 8beta-VE2 are two known synthetic selective ERbeta agonists, the biological activity of which have been tested in other mammalian cell models [25, 26], in comparison to the natural ligand 3beta-Adiol [11]. Rv1 cells were chosen as a model of primary androgen-responsive human PC; these cells have also the advantage to maintain an exponential growth up to 15 days* in vitro* and to display a steady doubling time for a long period [31].

As contrasting results are present in the literature on ERbeta expression in Rv1 cells [12, 33, 34], we assessed ERbeta gene and protein expression levels, which were shown to be low, but detectable. Interestingly, 3beta-Adiol at 10 nM, a dose that resembles endogenous intraprostatic levels [38], was found to be the sole ERbeta selective agonist active in decreasing cell proliferation both after short- (9 days) and long- (12 weeks) term intervals. Dose (10 nM) and antiproliferative effects of 3beta-Adiol appear similar to previously published data obtained in two commonly used CR-PC cells (DU145 and PC3 [39]) and in breast cancer cells [40].

This study shows for the first time that 3beta-Adiol efficacy persists over the time with a 20–40% reduction of cell proliferation during 12 weeks of administration. Although statistically significant at any time, such effect is particularly evident from 9 days to 5 weeks of administration and less pronounced over the following time frame (up to 12 weeks). This may be due to the possible development of some cell adaptive mechanisms, which, however, are not linked to drug resistance, as 3beta-Adiol is able to promote a progressive and significant increase of p21 protein expression.

The involvement of the endogenous ERbeta system in the mechanism of action of 3beta-Adiol in these cells is supported by the ability of the compound to activate ERE-mediated transcription, an effect that is dose-dependently counteracted by the presence of the pure antiestrogen ICI 182,780. Moreover, long-term administration of 3beta-Adiol and the consequent ERbeta activation are associated with a progressive increase of p21 expression levels and a slight decrease of the cyclin E (data not shown), suggesting a potential mechanistic relationship between these events. These findings appear to fit well with previous studies, demonstrating that activation of either the endogenous ERbeta in PC3 [41] and in DU145 cells [13] or of the stably transfected ERbeta in AD- or CR-PC cell lines [42, 43] results in the increase of p21 expression and cell cycle arrest.

During PC progression, PTEN inactivation is an established key modification for the emergence of androgen refractoriness [44]. Moreover, a partial loss of PTEN is extremely frequent in human primary cancers, particularly in PC, making the possibility to increase or maintain appropriate PTEN levels, an important target for chemoprevention. The ability of ERbeta activation to increase PTEN expression in cancer cells has been demonstrated by some authors in PC [45] and in other cancer models [42, 46, 47], but, to our knowledge, such effect of ERbeta activation over a long time frame has never been evaluated. To this regard, our results demonstrated that PTEN expression levels are not influenced by the activation of the endogenous ERbeta by 3beta-Adiol, as well as by 8beta-VE2, whereas DPN seems to display a slight activity from 5 weeks of treatment onward, which however influences cell proliferation only at the end of the chronic treatment (T12). These results suggest that in Rv1 cells the main target of ERbeta in the control of proliferation seems to be the modulation of cell cycle progression rather than inhibition of cell survival.

The androgen sensitivity of this cell line [33] is suggested by our results, demonstrating that the main constitutive active AR subtype (75 kDa) is much more expressed than the wild-type AR (110 kDa subtype). One of the criticisms for the use of 3beta-Adiol in PC cure is its potential retro-conversion to DHT [48] and the very recently suggested ability of the compound to bind also to ARs in some particular conditions [49]. The results here presented, demonstrating that the antiproliferative effect of 3beta-Adiol is still present in a PC cell model expressing functional ARs, are particularly important, because they suggest a possible use of 3beta-Adiol (or its analogs) also in the androgen-dependent phase of PC progression. Interestingly, AR subtype expression in Rv1 cells is not affected by chronic exposure either to 3beta-Adiol or to the two other ERbeta selective agonists (data not shown), excluding a possible contribution of AR-mediated cell proliferation over time.

Chronic DPN administration does not influence cell proliferation with the exception of the last time point evaluated (T12). As previously demonstrated in our laboratory, a 9-day exposure to DPN, at the same dose used in the present experiments, is able to significantly reduce DU145 cell proliferation [13]. Interestingly, the compound seems to be ineffective in LAPC-4 and in LNCaP cells, either in the absence or in the presence of DHT stimulation [50]. Notably, DU145 are CR-PC cells not expressing AR [51], while LAPC-4 and LNCaP cells are AD and express a 110 kDa AR [51]. Thus, it seems that, as opposed to 3beta-Adiol, the simultaneous presence of ERbeta and AR might interfere with DPN activity on PC cell proliferation. The possible interplay between the androgenic and estrogenic signaling pathways has apparently been evaluated only in breast cancer cells [52]. In this model, AR seems to target classical ERE sequences and it has been demonstrating an extensive interaction between AR and ERalpha in the control of target gene transcription, which results in a blunted proliferative action of ERalpha [52]. The possibility of a cross-talk between AR and ERbeta in controlling PC functions has never been studied yet but should be carefully examined in future studies to elucidate whether a similar mechanism could be active also in tumors where AR activation is the driving force for proliferation.

8beta-VE2 is as potent as estradiol in binding and activating ERbeta in prostate preparations [36, 37] and in inducing apoptosis in human prostatic basal cells [28]. However, the present study showed that this compound is completely unable to influence cell proliferation as well as p21 and PTEN expression in Rv1 cells. In a previously published study using different experimental conditions and cell models, 8beta-VE2, at a dose 60 times higher than that used in the present experiments and after a 12-h exposure, was able to activate the extrinsic pathway of apoptosis in another PC cell system, the PC3 cell line [27].

To get a better insight into the interactions of different ligands with the ERbeta pathway and the consequent biological effects (or lack of effect), we assessed the ability of 8beta-VE2 and 3beta-Adiol to induce ERE-mediated transcription upon binding either to the endogenous ERbeta in Rv1 cells or specifically to ERbeta1, transiently transfected into HEK293 cells (a cell line that lacks endogenous ERbeta proteins, [17]). 3beta-Adiol was able to activate ERE-mediated gene transcription in Rv1 cells, while 8beta-VE2 was completely inactive. On the contrary, 8beta-VE2 significantly and dose dependently stimulates ERE-mediated gene transcription through ERbeta1 in HEK293 cells, while 3beta-Adiol is completely ineffective in this experimental model.

One possible explanation of such opposite pattern of activation might be linked to a specific profile of expression of ERbeta subtypes in tumor cells and/or the different ability of the two compounds to bind to these subtypes and to activate transcription. It is indeed known that the alternative exon 8 present in the main ERbeta splice variants in humans (ERbeta2–ERbeta5) confers a conformational change in the second transactivation domain that alters the ability of the receptor to bind ligands and recruit cofactors [17]. However, all ERbeta subtypes can bind to canonical ERE-sequences on DNA as homo- or hetero-dimers [22]. It is also known that, during the development and progression of PC, ERbeta1 expression is gradually lost, while that of ERbeta2 and ERbeta5 increases [19]. Moreover, the relative expression between ERbeta2 and ERbeta5 differs among the different transformed prostate cell lines, since ERbeta2 is much higher than ERbeta5 in PC3 cells, while the opposite pattern is present in LNCaP cells [22].

The assessment of the relative mRNA expression levels of the three main ERbeta subtypes present in the human prostate (ERbeta1, -beta2, and -beta5) in Rv1 cells indicates that these cells possess low levels of ERbeta1 and higher levels of ERbeta2 and ERbeta5 (3- and 5-folds versus ERbeta1, resp.). In the Western blotting experiments, using an antibody able to recognize the N-terminus common to all the ERbeta subtypes (Figure 2(b), multiple bands within the 50–60 kDa range are shown: the presence of these bands is in agreement with the results obtained by qPCR and consistent with those reported by Leung et al. [17].

Differences in the cell response to ERbeta selective ligands among Rv1 (present results), DU145 [14], and PC3 cells [27] might be related to a different expression pattern of the ERbeta subtypes in the three cell lines. To test the possibility that 3beta-Adiol and 8beta-VE2 show a different transcriptional activity upon binding to ERbeta1, ERbeta2, or ERbeta5, we overexpressed the three ER subtypes alone or in combination in HEK293 cells and test the transcriptional activity of the two compounds by an ERE-coupled reporter gene. 8beta-VE2 was unable to stimulate transcription in the presence of ERbeta2 or ERbeta5 alone, confirming the inability of these ER subtypes to activate ERE-mediated transcription* per se*. On the contrary, the compound significantly stimulated gene transcription when ERbeta wild type was present, giving the possibility to form ERbeta1 homodimers or to heterodimerize with the other subtypes (ERbeta1:beta2 and ERbeta1:beta5). Analogous cotransfection experiments have demonstrated that, in comparison to ERbeta1, the coexpression of ER subtypes beta1:beta2 and of beta1:beta5 significantly increase the transcriptional activity of estradiol as well as other xenoestrogens [17]. In agreement with these findings, in our experiments, the transcriptional activity of 8beta-VE2 is significantly higher in the presence of the heterodimer beta1:beta5 in comparison to ERbeta1 alone. However, as opposed to what presented by these authors, we did not find significant difference between ERbeta1 alone and the dimer beta1:beta2. One possibility to explain the different behavior of 8beta-VE2 might be the propensity of the various ERbeta agonists to promote ERbeta homo- or heterodimerization. In line with this hypothesis, also phytoestrogens appear to favor only ERbeta1 homodimerization [17].

In agreement with the previous results, 3beta-Adiol appeared to lack any transcriptional activity in ERbeta transfected HEK293 cells in the presence either of a single or of different receptor subtypes. It should be underlined that in our experiments we cotransfected equimolar amounts of receptor subtypes, while, in normal or neoplastic prostate cells, the relative expression levels are widely variable ([19] and the data here presented). Thus, the possibility that 3beta-Adiol might stimulate ERE-mediated transcription only in the presence of a peculiar ratio among the different ER subtypes cannot be ruled out. As previously mentioned, the synergistic effect of ERbeta2 and ERbeta5 on ERbeta1-mediated transcription depends on the ligand used. If ERbeta subtypes are expressed at different levels during the natural history of PC progression, this peculiar pattern, forming a wide and plastic array of homo or hetero-dimers, may contribute to the different pharmacology of the ERbeta selective agonists. The presence of a functional AR system in Rv1 cells adds a further level of complexity and might explain the different behavior between 3beta-Adiol and the other synthetic compounds. If part of the 3beta-Adiol, through its retro-conversion to DHT, binds to AR [48, 49], the effects observed on cell proliferation and p21 expression might imply a cross-talk between AR and ER signaling pathways and the recruitment of a particular set of coregulators. As previously mentioned, AR can also target classical ERE sequences and may interact with the ER systems, as already shown in breast cancer cells [52]. Studies are in progress in our laboratory to evaluate this hypothesis.

## 5. Conclusions

The results presented in this paper by using different ERbeta selective agonists demonstrate that the activation of the ERbeta pathway has an antiproliferative effect also in androgen-responsive primary PC tumors and that this activity is maintained for a long period of time. In addition, from our results it clearly appears that the mechanism of action through which ERbeta controls prostate cell proliferation is still obscure in some aspects because it possibly implies a complex interplay among ERbeta subtypes (which depends on their peculiar pattern of expression) and/or an interaction with the AR system. The high variability of ERbeta subtype levels in normal, preneoplastic, and cancerous prostatic cells, including Rv1 ([19] and the data shown here), coupled to the different behavior of selective ERbeta agonists on PC cell functions (as appears from our studies), strongly suggests that a careful assessment of the expression pattern of the ERbeta subtypes should not be disregarded, when considering ERbeta-targeted new drugs for PC chemoprevention.

Moreover, if different ERbeta selective agonists might produce distinct biological and clinical effects, it cannot be assumed that the lack of effect of one compound* in vitro* or in clinical trials may be extended to all the chemical classes of compounds that bind to ERbeta. In line with this concept, to prevent and/or slow down PC progression through ERbeta activation, it should be very important to identify the specific outcomes of the different ERbeta selective ligands in a PC specimen of each single patient. This strategy will be helpful to choose the appropriate drug for the therapy.

## Figures and Tables

**Table 1:** 

Target gene	Forward primer	Reverse primer
ERbeta1	GTCAGGCATGCGAGTAACAA	GGGAGCCCTCTTTGCTTTTA
ERbeta2	TCTCCTCCCAGCAGCAATCC	GGTCACTGCTCCATCGTTGC
ERbeta5	GATGCTTTGGTTTGGGTGAT	GGAGGAGTGGGTGTCGCTGT
Beta-actin	CCACCATGTACCCTGGC	CGGACTCGTCATACTCCTGC

**Table 2:** 

Target protein	Primary antibody	Secondary antibody
ERbeta (all)	Ab288 (Abcam, Cambridge, UK); 1 : 500 dilution	WesternDot 625 detection kits (Life Technologies Italia, Monza, Italy); 1 : 2000 dilution

AR	Sc816 (Santa Cruz Biotechnology, Santa Cruz, CA, USA); 1 : 400 dilution	HRP-conjugated anti-rabbit (Santa Cruz Biotechnology, Santa Cruz, CA, USA); 1 : 2000 dilution

PTEN	ab32199 (Abcam, Cambridge, UK); 1 : 1000 dilution	HRP-conjugated anti-rabbit (Santa Cruz Biotechnology, Santa Cruz, CA, USA); 1 : 8000 dilution

p21	05-345 (Millipore, Billerica, MA, USA); 1 : 1000 dilution	HRP-conjugated anti-mouse (Santa Cruz Biotechnology, Santa Cruz, CA, USA); 1 : 5000 dilution

Tubulin	T9026 (Sigma-Aldrich, Monza, Italy); 1 : 2000 dilution	HRP-conjugated anti-mouse (Santa Cruz Biotechnology, Santa Cruz, CA, USA); 1 : 8000 dilution

Beta-actin	Sc1616 (Santa Cruz Biotechnology, Santa Cruz, CA, USA); 1 : 4000 dilution	HRP-conjugated anti-goat (Santa Cruz Biotechnology, Santa Cruz, CA, USA); 1 : 4000 dilution
